# Obesity Is a Marker of Reduction in QoL and Disability

**DOI:** 10.1100/2012/167520

**Published:** 2012-03-12

**Authors:** Anna Sirtori, Amelia Brunani, Valentina Villa, Maria Elisa Berselli, Marina Croci, Matilde Leonardi, Alberto Raggi

**Affiliations:** ^1^Department of Internal Medicine, Istituto Auxologico Italiano IRCCS, Piancavallo, 28824 Verbania, Italy; ^2^Clinical Psychology, Istituto Auxologico Italiano IRCCS, Piancavallo, 28824 Verbania, Italy; ^3^Department of Medical Sciences and Rehabilitation, Istituto Auxologico Italiano IRCCS, 20145 Milan, Italy; ^4^Neurology, Public Health and Disability Unit, Neurological Institute C. Besta IRCCS Foundation, 20133 Milan, Italy

## Abstract

The purpose of this paper is to verify the association between outcome measures of health-related quality of life (HRQoL) and disability, BMI, gender, and age. Adult obese patients were clustered using HRQoL (IWQoL-Lite) and disability (WHO-DAS II) scores into three groups: mild, moderate, and high. One-way ANOVA with Bonferroni post hoc test was used to evaluate differences in age and BMI between subjects from different clusters, contingency coefficient to test the relationship between cluster groups and gender. In total, 117 patients were enrolled: subjects with higher disability and HRQoL decrement were older and had higher BMI. Women were more likely to present moderate disability and reduction in HRQoL, while men more likely presented mild disability and HRQoL reduction. Our data further confirm the connection between disability and HRQoL, high BMI and older age. These data obtained with outcomes measures might better address rehabilitation programs.

## 1. Introduction

The World Health Organization [1] rates obesity as the 5th leading global risk factor for mortality in the world with 2.8 million deaths (4.8% of global death) and 10th for global burden of disease. With regard to mortality, the results of a recent report that used data derived from 19 prospective studies with 1.46 million white adults and a wide range of body mass index (BMI) were impressive: they showed that hazard ratios for all-cause mortality was up to 2.51 in patients with BMI higher than 40 kg/m² and was 4.42 for deaths due to cardiovascular diseases in the same group of subjects, also restricted to healthy participants who never smoked [2]. 

However, obesity cannot only be seen as a risk factor for mortality. It is in fact a chronic disease that produces an increase in morbidity and dependence on others for daily needs and is responsible for the loss of healthy life years, estimated in 2.3% of disability-adjusted life years (DALYs) [1]. Obesity as an impact of patients' live as it affects disability-free life by reducing it to 2.7 years in men and 3.6 years in women and, at the same time, by increasing the whole duration of disability to 2.0 years in men and 3.2 years in women [3] meaning that obesity-related disability increased in conjunction with declining mortality rates. Recently, the National Health and Nutrition Examination Surveys (NHANESs) analyzed data from two periods (1988–1994 and 1999–2004) [4]. Results show that, compared to normal-weight subjects, persons with mild obesity had twice the odds of daily life activities (ADLs) limitations (OR: 2.11; 95% CI: 1.15–3.86), while those with severe obesity had four times (OR: 3.96; 95% CI: 1.79–8.79). Furthermore, between NHANES I and NHANES II, the OR for obese persons, compared to normal-weight subjects, increased by a factor of 1.56 between time 1 and time 2 (95% CI: 1.03–2.36) therefore, a prolonged duration of obesity determined an further increase in the likelihood of having limitations in ADLs.

The most relevant issues responsible for health deterioration in obese subjects, and their connection to obesity degree and age, are not completely clear yet [5]. The results of a European population study (SHARE: Survey of Health, Ageing and Retirement in Europe) were reported in a paper describing the health correlates of obese subjects aged 50 years and over [6]. Results showed that, compared to normal-weight subjects, obese persons had between 2 and 2.4 the odds of reporting health complaints, between 2.4 and 2.7 the odds of reporting two or more chronic diseases, and between 0.4 and 0.5 the odds of self-reporting good to excellent health. In addition to this, obese men also reported between 1.6 and 2.4 the odds of having a physical disability, while women reported even more disability, as they reported between 2.1 and 3.5 the odds of physical disability compared to nonobese women.

It is likely to suppose that the increase of musculoskeletal and joint problems and the association with other chronic diseases might explain mobility limitations (e.g., bathing, dressing, getting in or out of bed, walking, climbing stairs, raising from a chair), early fatigue, dyspnoeas, and a reduction in different kinds of job tasks [7]. The issue of mobility limitations is one of the most studied: a recent literature review evidenced a clear relationship between increasing obesity severity and reduced mobility, both in cross-sectional and longitudinal perspective, and gender differences accounting for higher limitations in women than in men [8]. Obesity produces important effects also in psychological symptoms—such as negative self-evaluation, decreasing self-image, anxiety, and depression—which in turn determine reduced social activities [9]. An important role for the development of psychological problems is played by the weight-based stigmatization associated with higher BMI that obese persons suffer from [10, 11] and is experienced as a negative stereotype, prejudice, and discrimination, reported in the areas of employment, education, health care, and media as well as interpersonal relationships [12].

Taken together, these features related to impairments in physical and psychological functions and limitations in daily life constitute the profile of functioning and disability of obese subjects. Disability is defined by WHO with its International Classification of Functioning, Disability and Health (ICF) [13], as the negative interaction, experienced by an individual with a health condition, between impairments at the body level and presence of barriers in the environment. Such a conceptualisation recognises that disability is not an intrinsic feature of an individual, but is also experienced and influenced by the environment in which the person lives. Obese patients' profile of functioning has been evaluated, using ICF-based methodologies, in two previous papers [14, 15]. The first reported the areas in which difficulties are reported, showing that areas connected to mobility and self-care are those most frequently reported as being limited. The second showed that impairments at the level of the body are much more closely related to limitations in performing activities than the effect of environmental factors.

Patient-derived outcome measures importance is increasingly recognised. Among these measures, health-related quality of life (HRQoL) is one of the most evaluated in patients with chronic conditions. In the field of obesity research, the evaluation of HRQoL was recognised by the United States Task Force on Developing Obesity Outcomes and Learning Standards [16] (TOOLS), which recommended the use of SF-36 health-related quality of life and its short form (SF-12) as a generic HRQoL measure in obesity [17]. Recent studies reported that increased body weight corresponds to a deterioration in the domain of physical functioning and general health score, particularly in women, while deterioration was less evident in mental functioning [18, 19]. Generic measures, however, do not address key domains relevant to obesity. Obesity-specific measures have been developed, including the impact of weight on quality of life (IWQoL), 74-item measure later reduced to the IWQoL-Lite of 31 items [20], that better target obesity-specific issues [21]. The utilisation of patient-derived outcome measure, in addition to clinical outcome such as weight loss, is relevant to understand or prevent the social disadvantages associated with obesity and its stigmatization. In a previous study, Sirtori and colleagues analyzed the relationship between HRQoL, disability, and obesity, underlying the importance of evaluating both HRQoL and disability in obese patients undertaking rehabilitative intervention, as the two outcome measures underline different and not transposable dimensions, thus reporting complementary information [22].

The evaluation of outcomes in rehabilitation is strictly dependent on both the objectives of intervention as well as on the levels of disability and HRQoL that a patient displays. However, the effect of body weight on HRQoL and disability, measured according to ICF's biopsychosocial model, is not systematically evaluated. The relationship between increased BMI and functional limitations is of primary relevance, in particular in consideration with ageing trajectories. In fact, as reported by the results of a paper focussed on a large UK ageing study in which functional limitations were compared across subjects with different BMI groups, the excess of body weight in aged persons is associated with greater risk of impaired physical functions [23]. What is lacking is an information on the degree of association between outcome measures—which enable to identify subjects with different degrees of disability and HRQoL reduction—and the severity of obesity, as well as sociodemographic variables such as sex and age. The identification of these relationships is of primary relevance to enable researchers and policy makers to face the challenge of the increasing burden of obesity-associated disability, health, and social costs.

## 2. Material and Method

### 2.1. Study Design

In this cross-sectional observational study obese inpatients and outpatients under rehabilitation were consecutively enrolled at the Auxologic Institute in the period between June 2009 and May 2009. Patients were included if their BMI was higher than 30 and if they were at least 18 years old. Patients unable or not willing to participate in a 30-minute interview were excluded. Those who agreed to participate signed an informed consent form approved by the institute's Ethical Committee.

### 2.2. Instruments

To evaluate HRQoL, the Italian version of short form of impact of weight on quality of life (IWQoL-Lite) questionnaire [20] was used. This instrument is a well-designed, validated, and responsive 31-item self-administrated measure of weight-related quality of life. It was developed for the purpose of assessing baseline, group differences and measuring changes in HRQoL for persons being treated for obesity. IWQoL-Lite investigates five domains (physical function, self-esteem, sexual life, public distress, and work) related to obesity and provides a total score (sum of scale scores). Participants are asked to rate items with respect to the past week on a five-point scale (from “never true” to “always true”). The total score ranges between 31 and 155, with higher scores reflecting poorer quality of life.

To evaluate disability, the WHO-DAS II (World Health Organization Disability Assessment Schedule II) [24, 25] was applied. The WHO-DAS II is an ICF-based structured interview composed by 36 items and captures an individual's level of functioning and disability that is reliable and applicable across cultures in adult populations. The WHO-DAS II is used for many purposes: for conducting population surveys, for registers, and for monitoring individual patient outcomes in clinical practice and in clinical trials of treatment effects. It takes around 15 minutes to be administrated and cover six domains: understanding and communicating (6 items), getting around (5 items), self-care (4 items), getting along with people (5 items), life activities, divided into household (4 items) and work/school (4 items), and participation in society (8 items). Patients are required to answer questions regarding how many difficulties they had in the last 30 days due to their health condition on a five-point scale from 1 (no difficulty) to 5 (extreme difficulty or cannot do it). Both subscale score and global scores are available. Global scores are calculated on the basis of all 36 items or of 32 items in case respondents do not complete the section related to work/school. Scores range from 0 to 100, with higher scores reflecting greater disability.

### 2.3. Statistical Analysis

A K-Means Cluster Analysis was performed in order to group patients on the basis of IWQoL-Lite and WHO-DAS II global scores, and one-way ANOVA was used to evaluate cluster centroids. We predefined 3 clusters to identify high, moderate, and low disability and HRQoL decrement, without specifying initial cluster centroids.

One-way ANOVA with Bonferroni post hoc test was used to evaluate differences in age and BMI between subjects from different clusters. Contingency coefficient was employed to evaluate strength and significance of the relationship between cluster groups and gender.

Statistical significance was set to be *P* < .05 and 2-tailed testing for one-way ANOVA. Data analysis was performed using SPSS 11.0.

## 3. Results

One hundred and seventeen obese patients (80 females, mean BMI 43.7 kg/m², SD 7.0) aged between 19 and 81 yrs (mean 47.4 yrs, SD 14.8) were enrolled. According to WHO classification 12.3% of patients had mild (BMI between 30 and 34.9), 26.5% moderate (BMI between 35 and 39.9), and 61.2% severe obesity (BMI higher than 40).

The results of K-Means Cluster Analysis are reported in Table 1. Patients have been divided into three groups on the basis of WHO-DAS II and IWQoL-Lite global scores: cluster *A* is composed of 43 patients (36.8%) with lower disability and lower reduction in HRQoL; cluster *B* is composed of 50 patients (42.7%) with moderate disability and intermediate reduction in HRQoL; cluster *C* is composed of 24 patients (20.5%) with higher disability and higher reduction in HRQoL. Both of the scales provided significant contribution to the identification of cluster membership, as showed by high *F* values.

The results of one-way ANOVA are reported in Table 2. Bonferroni post hoc test shows significant differences, for both age and BMI, between patients in the cluster with higher disability and HRQoL scores and those in the clusters with lower and intermediate scores, while no difference was observed between the latter two clusters.

With regard to the association between gender and cluster membership, 21 males out of 37 (56.8%) were in cluster with lower HRQoL and disability scores, 12 (32.4%) in that with intermediate scores, and 4 (10.8%) in the cluster with higher scores; 38 out of 80 females (47.5%) were in the cluster with intermediate scores, 22 (27.5%) in that with lower, and 20 (25%) in that with higher scores. Contingency coefficient was 0.277 (*P* = .008).

## 4. Discussion

The aim of this study was to evaluate the degree to which groups of obese patients, defined on the basis of disability and HRQoL (low disability and HRQoL reduction, moderate disability and intermediate HRQoL reduction, and high disability and HRQoL reduction) also report different BMI, age and are differently composed by males and females. Results show that subjects with higher disability and HRQoL decrement also have higher BMI and older age and also highlight a gender-based association: males were in fact more likely to express low disability and HRQoL decrement, while females were more likely to be in the intermediate group.

The impact of obesity on HRQoL deterioration has been reported in some previous studies that also evaluated the presence of associated problems like difficulties in mobility, pain, sleep quality, physical functioning and social functioning, and general psychopathology  [26–29]. In particular, subjects with higher BMI report lower scores in physical—but not mental—aspects of HRQoL [30], and in a wide cross-sectional study, higher BMI, higher age, and higher numbers of current somatic and mental disorders were found to negatively predict the physical dimension of HRQoL [31]. Controlling for comorbidities, such as osteoarthritis, asthma, and diabetes does, however, reduce but not eliminate the reduction of HRQoL associated with obesity [32]. At the same time, it cannot be ignored that presence of obesity is also associated with an increased likelihood to suffer from chronic conditions, reporting more health, complaints, having a worse perception of one's own health, and reporting higher disability [6]. Moreover, the decrease in HRQoL or other psychological problems (depression or anxiety) is the key element that might increase the risk of disability. In fact a 12-years cohort study recently reported that patients with obesity and repeated distress had a double risk of disability [33]. Our results are in line with those previously reported and provide new insights as the procedure we employed is not based on “known groups” based on BMI, but rather on outcome measures. Such approach enabled us to define the severity of problems experienced by patients independently from other clinical or sociodemographic features. The added value lies in the possibility to understand the association between subjectively reported information, dealing with the difficulties in daily lives and with the appreciation of health-related quality of life, and relevant variables that are usually considered as independent ones. This study provides further confirmation of such relationship, as the outcome variable is here used to create groups of patients that are likely to experience different levels of health-related problems due to obesity. In a previous study, Sirtori and colleagues [22] demonstrated that IWQoL-Lite and WHO-DAS II evaluate different and not transposable psychosocial facets and that these seem to follow a specific trend: high disability is associated with a reduction of quality of life, and low disability is associated with a better quality of life. The results of the present study added information on age and gender. In fact, patients with high level of disability and severe reduction of quality of life were significantly elder, in addition to having a higher BMI. These results are in line with what is reported in a recent literature review, which highlighted that elevated BMI is a risk factor for physical disability among older adults [34], and with an NHANES study that showed that indicators of obesity such as BMI and waist circumference are related to different indicators of disability in older adults [35]. In our opinion, it is important to evaluate both HRQoL and disability as these measures provide information on several areas (self-care, self-esteem, sexual life, daily life activities in house or work, communication, and public distress), thus identifying target areas of rehabilitation interventions.

In line with other studies [36], our data show that obese women seem to have more probability to present moderate disability and reduction in HRQoL than men. Probably in our study, as in general population, obese women present a greater susceptibility than men such as lower-extremity mobility, lower bone mineral density, and high risk fracture. However, mobility limitations among obese women seem to be an established fact, as reported in a recent literature review [8], and are likely to be the most relevant contributors to disability as a whole in obese subjects. Besides, obesity is known to be associated with increased prevalence of common mental disorders (e.g., depression and anxiety), and there is evidence of gender differences in this relationship: obese women seem to have more probability to suffer a mood or anxiety disorder than obese men [37, 38]. Probably these mental conditions could have a significant role in women's worst HRQoL and disability, highlighted in our study.

Two limitations to study results' generalisation should be taken into account. The first lies in the cross-sectional design, which does not enable to define causal relationships. The second lies in sample composition, which is relatively small, constituted by patients attending to a specialty centre only, and with an overrepresentation of those with BMI >40.

In conclusion our study shows that there is a connection between increased disability and quality of life and increased BMI. Considering the increasing prevalence of obesity and population ageing in western societies, data and methodologies showing the impact of the condition on persons' functioning are needed, as they could enable to provide clear data on the effect of treatment on health and health-related outcomes.

## Figures and Tables

**Table 1:** K-Means Cluster Analysis: patients were divided into three groups according to WHO-DAS II and IWQoL-Lite scores.

	Cluster			*F*
	*A*	*B*	*C*	
WHO-DAS II	8.6 (6.1–11.1)	22.7 (19.6–25.9)	47 (42.3–51.8)	110.6*
IWQoL-Lite	59 (55–62)	87 (84–90)	110 (104–117)	161.1*

*Note.* For each group, cluster centroids and 95% confidence interval are reported; *P < .001. Bonferroni post hoc test was significant for each pair, both for WHO-DAS II and IWQoL-Lite total scores. Cluster *A*: 43 cases, lower disability, and HRQoL reduction; Cluster *B*: 50 cases, intermediate disability, and HRQoL reduction; Cluster *C*: 24 cases, higher disability, and HRQoL reduction.

**Table 2:** Differences in age and BMI among patients in different clusters.

	Cluster			*F*	*P* value
	*A*	*B*	*C*		
Age (years)^a,b^	44.3 (39.7–48.8)	46.0 (41.8–50.2)	55.9 (50.9–60.9)	5.54	*P* = .005
BMI (kg/m²)^a,b^	42.1 (40.1–44.0)	43.0 (41.0–45.0)	47.8 (45.2–50.4)	6.06	*P* = .003

*Note.* ^a^Bonferroni post hoc test significant at *P* < .05 between patients in Cluster *A* and Cluster *C*; ^b^Bonferroni post hoc test significant at *P* < .05 between patients in Cluster *B* and Cluster C.
